# A Participatory Community Diagnosis of a Rural Community from the Perspective of Its Women, Leading to Proposals for Action

**DOI:** 10.3390/ijerph18189661

**Published:** 2021-09-14

**Authors:** Maria Jose Alberdi-Erice, Homero Martinez, Esperanza Rayón-Valpuesta

**Affiliations:** 1 Facultad de Medicina y Enfermería, Sección Donostia, Universidad Pública del País Vasco (UPV), 20014 San Sebastián, Spain; mariajose.alberdi@ehu.eus; 2 Hospital Infantil de México Federico Gómez, Ciudad de Mexico 06720, Mexico; 3 Nutrition International, Ottawa, ON K2P 2K3, Canada; 4 Facultad de Enfermería, Fisioterapia y Podología, Universidad Complutense de Madrid (UCM), 28040 Madrid, Spain; erayon@ucm.es

**Keywords:** community diagnosis, participant observation, ethnography, health assets, rural health, social determinants of health, nursing, community health, environmental health, participatory research

## Abstract

In primary health care, a community diagnosis is necessary to provide a detailed description of the community as well as an evaluation of the community’s health, including the main factors responsible for it and the needs felt by the population. This article presents a community health diagnosis following a participatory design, taking the perspective of women living in the community, to identify proposals for action. An ethnographic study was carried out in the community of Mañaria (Spain), using semi-structured interviews, in-depth interviews, key informants, participant observation, desk review, and photography. A sample of 21 women were interviewed until reaching saturation of the information. This information was complemented by that provided by five key informants. Data analysis included text analysis, coding, and categorization. Preliminary results were presented to the informants for validation and further refinement, and proposals for action were identified and followed up. Six categories were identified, representing different areas of intervention: population, jobs and economy, public and private spaces, lifestyles, processes of socialization, and health care assets. For each of these areas, the main problems were identified, as were the health care assets and proposals for action. The community diagnosis has been shown to be useful not only to identify health needs but also as an efficacious instrument to trigger social and public health actions that may be undertaken at the institutional level.

## 1. Introduction

In primary health care, carrying out a community diagnosis provides the necessary background for any intervention. This diagnosis is meant to provide a detailed description of the community as well as an evaluation of the community’s health, including the main factors responsible for it and the needs felt by the population [1]. There is ample consensus in pointing out that the proposed interventions must respond to the specific needs expressed by the community, which requires working together to identify these needs and how to address them, taking into consideration the different factors that influence the community and developing models and strategies that allow the best approaches [2,3,4]. When successfully implemented, actions that were identified as part of the community diagnosis help strengthen ties between different actors in the community (i.e., neighbors, those working in public health services, institutions, etc.). This, in turn, represents an opportunity for the community to develop leadership and empowerment, facilitating action-oriented enterprises [5]. It is highly relevant for any community process, such as a community diagnosis, to reach a conclusion with an evaluation and analysis of how effective the process was [6,7].

The conceptualization of health on which this study was based is that health is a universal right and a dynamic, subjective, and individual experience of a person who lives in a community sharing economic, social, and cultural characteristics and affected by environmental factors and rules for living together. Community health is understood as the collective expression of the health of all individuals and families, the social, cultural, and environmental characteristics, as well as health services and the influence of social, political, and global factors—that is, the social determinants of health [7].

For these reasons, the conceptualization of the present study was based on a comprehensive paradigm, as we characterized the community by including the social determinants of health identified in the community and then pictured the day-to-day life of people in the community with a holistic approach, including a positive and multidimensional vision of health [8,9].

A community approach to health is, by definition, participatory. In this sense, the health diagnosis is a community process, and as such it requires participation of three main actors: administration (local or higher level); technical and professional resources—people who are directly related to the population on a daily basis and who manage services, programs, and benefits (educational, social, sanitation, economic, etc.); and citizens, involving social organizations (associations and formal or informal groups) and other actors who are active participants in the whole process, through their daily active participation in public life [6,7,10].

Including women in this process will allow for better protection, promotion, and self-care, both for women as well as for the community at large, by identifying mechanisms that promote dialogue, agreement, and negotiation between community members and institutions. Including women will also recognize their highly valued roles in maintaining current and ancestral knowledge as well as cultural attitudes toward health systems [11,12]. Although some studies consider women’s roles in community diagnoses [13,14,15], discussion of their participation is often limited to their role as informants, with little or no participation in decision making. Studies that include community participation in health diagnoses are scarce. Looking specifically at the Spanish context, there are some relevant examples. A study carried out in Ronda, Malaga, aimed to understand the perceptions of citizens about factors that influence well-being and quality of life of the community at large, going beyond an individualistic model [16]. A second example, focused on improving quality of life, took place in the neighborhoods of Las Remudas and La Pardilla, in Las Palmas de Gran Canaria. The community diagnosis was jointly undertaken by those who lived, worked, and managed services in these adjoining neighborhoods, providing the foundation for programing the required actions to improve the whole area [17]. A third example comes from Barcelona [5], where mixed methods were used to obtain a community diagnosis of the most marginalized neighborhoods in the city. A desk review was complemented by focal groups, nominal groups, and individual interviews, allowing for a holistic appraisal of needs and potential solutions. Looking at other countries, examples can be found in Latin America [18,19] and in Europe, including those associated with the Healthy Cities project that took place in Europe at the end of the 1980s/beginning of the 1990s [20]. These studies were focused mainly on identifying priorities for intervention that stemmed out of these diagnoses but had no follow-up or evidence regarding their actual implementation in the medium or long term [21,22]. Furthermore, in none of these instances was the gender perspective incorporated into the process.

The objective of the present work was to carry out a community diagnosis from the perspective of women living in the community, based on their experiences, beliefs, values, and opinions [13,23], to identify the social determinants of health that could be modified to improve the overall health of the community and identify the corresponding proposals for change.

## 2. Materials and Methods

The design corresponded to a community diagnosis following community-based participatory research methods [2], followed by qualitative data collection and interpretation based on ethnographic methods [24,25]. This allowed the researchers to provide context for the community as well as its history, language, and general characteristics. It also allowed for the collection of individual information within this context and thus description and interpretation in greater depth of the community’s culture, values, beliefs, and behaviors [23,26].

The community selected for the study was Mañaria, located in the central-southeastern part of the Historical Territory of Vizcaya, in northern Spain. At the time of the study, the community had 522 inhabitants, distributed in three types of settlements: urban center, five neighborhoods, and dispersed houses (caseríos), which is typical of the region. The structure of the female population in Mañaria during 2009–2011 and the sample selected for the present study (2008) is shown in Table 1.

**Figure 1:**
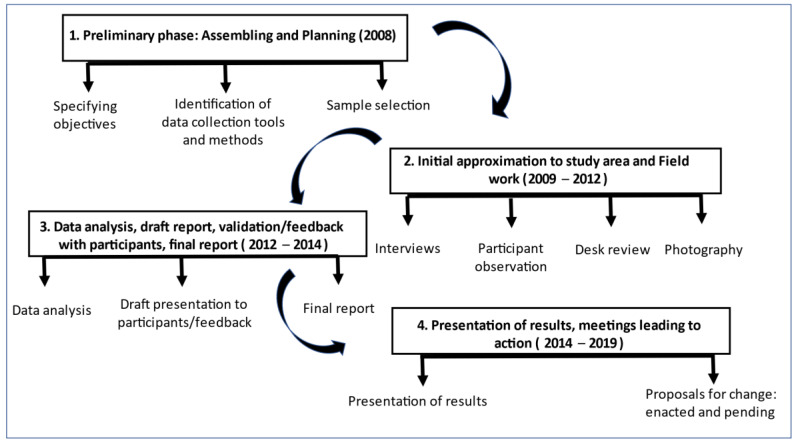
Phases of fieldwork (2008–2019).

Fieldwork took place in two phases, conducted in 2009 and 2014. Once data collection and analysis were complete and the main results were summarized, the researchers went back to the field to present results to the community and identify proposals for action, to address challenges, improve negative aspects, and improve or enable positive findings. This phase was so well received that the research team was encouraged to present their proposals for action to the rest of the community and the local authorities, leading to the approval of several of these proposals, which were implemented by the end of the study (as will be discussed). This study was completed in 2019. Figure 1 describes all phases of the study (2008–2019).

The sample included 21 participants, identified through purposive sampling. Researchers considered the availability of women to participate in qualitative interviews and respond to the sociodemographic questionnaire, seeking to maximize variability in terms of socio-demographics, age, and geographical location [27]. Additionally, five key informants were selected, to include women that were well informed about the specific topics the researchers were interested in, who were easy to approach, and who could help to better understand these topics [28]. For this purpose, the researchers identified women who were employed as professional staff in medical services, either as a physician or a nurse, as well as social agents who worked directly in the field in small environments, promoting interaction and health among community members. These participants contributed their “network experience” to the study [29]. As data collection progressed, sampling stopped once saturation of the responses was reached. Saturation was reached when subsequent interviews produced no more relevant new data [24].

Data collection methods included participatory observation, in-depth interviews, and semi-structured interviews. There was also a desk review, consulting multiple sources and complemented by photography [24,30].

Our initial approach was to conduct exploratory, open-ended, in-depth interviews with every informant; in two cases, the researchers resorted to a second in-depth interview to get a deeper understanding of emerging topics and help guide interpretation. In the end, there were 28 in-depth interviews, lasting an average of ~1 h each. These in-depth interviews allowed the research team to reach implicit reasoning and to identify values and beliefs around health and its social determinants, expressed in discourse and experiences narrated by these informants. Open-ended interviews identified topics that were later explored in a more systematic way through semi-structured interviews (Table 2). Semi-structured interviews were useful for collecting information and opinions following the theoretical constructs of the integral health paradigm mentioned earlier. These provided the conceptual framework from which the proposed categories were extracted (Table 2).

On a first approach, interviewees were asked general questions like “What aspects do you like about this community?”, “What things do you miss?”, “What aspects do you think could be improved?”. Next, we moved on to explore aspects more specifically related to community health, like “What is your opinion about community health in Mañaria?”, “What aspects may contribute so that this community is more healthy or less healthy?”.

Interviews were carried out in the two languages widely spoken in the community, Spanish and Euskera, the autochthonous language. Use of the autochthonous language allowed for more fluid communication with informants who used it habitually, as it helped build rapport and empathy, both highly valued in qualitative interviewing [24].

Participatory observations took place at different times and places. This allowed researchers to capture the variability in behaviors and discourse that are directly or indirectly related to health and to get to know and understand the context in which these took place, thus facilitating the understanding of meanings and their interpretation. During fieldwork, one of the authors (MJA) lived in the community, further facilitating participatory observation.

**Figure 2:**
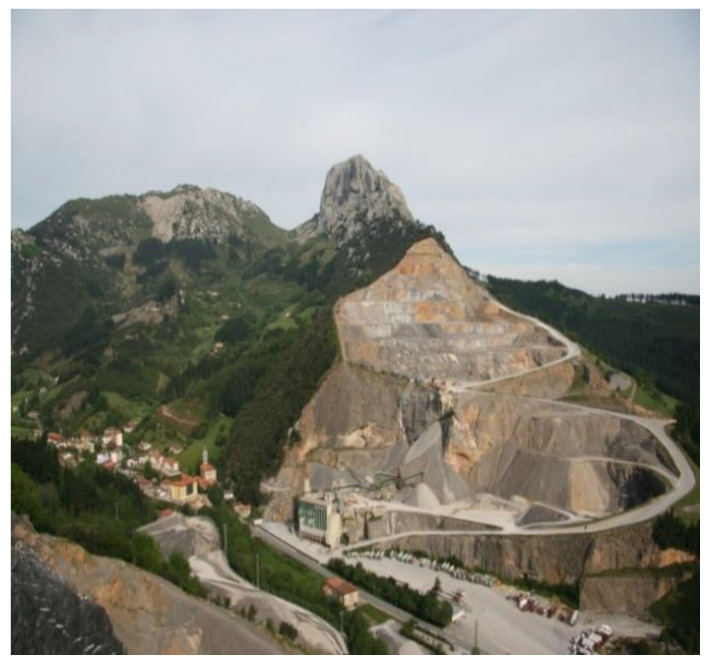
Markomin-Goikoa quarry in Mount Mugarra.

All observations were documented in a field diary [24], which was often enriched by photographs of places observed (as shown in Figure 2) [30]. These methods helped us better understand the context and were useful at the time of data interpretation among the whole team, e.g., when visualizing the impact of local quarries, or the effect of auzolana, a type of community work where neighborhood residents engage in collaborative work without pay on activities meant to benefit the community. Likewise, these methods were useful for supporting triangulation of information during data analysis.

All interviews were audiotaped and transcribed before proceeding to text analysis. Manual coding preceded coding and categorization, followed by grouping and comparison between categories. These were constructed based on similarities and affinity, following the inductive method proposed by Glaser and Straus [31], which allowed us to propose meta-categories and topics or thematic nuclei. This step in data analysis involved discussion and agreement between investigators and was strengthened by data triangulation and control for potential biases. The analysis covered three stages: coding and identification of categories for analysis, grouping in larger categories or meta-categories, and identification of thematic nuclei.

After the research team produced draft results from the initial analysis, these were presented to the 26 informants, seeking validation and fostering further discussion to help fine-tune or clarify any aspect of the researchers’ interpretation. This not only allowed for some rectification but also allowed researchers to collect new information, to understand nuances, and to include participants in the process, thus giving them a true sense of ownership of their own reality [22,32]. Once there was consensual agreement on the final draft, results were presented at the Town Hall, followed by public debate that led to specific commitments by political and sanitation authorities. This activity further reinforced women’s empowerment.

The research design took into consideration ethical considerations as recommended by the Helsinki Declaration; it was approved by the Ethical Committee of Universidad Pública del País Vasco-Euskal Herriko Unibertsitatea and followed the principles of the Declaration of Helsinki [33]. All interviews required oral consent from participants, and audio recordings followed informed consent, anonymity, and confidentiality protocols. All transcribed information was de-identified, using only letters/numbers for the purposes of coding. To ensure the soundness of the research, the COREQ guidelines [34] were followed as well as criteria proposed by Calderón [35]: validity, adequacy, relevance, and reflexivity.

## 3. Results

### 3.1. The Sample

The sample included 26 women, five of whom were key informants. The mean age was 47 years, with the following breakdown (sample) by age group: 10–24 years (*n* = 2), 25–54 years (*n* = 12), 55–69 years (*n* = 5), and >70 years (*n* = 2). Twenty-three women were permanent residents in the community; three did not live there but worked full time in the community. All geographical areas of the community (urban center and five neighborhoods) were represented by one or more women in the sample.

Four key informants were employees of the three main government institutions in the community: town hall, social services, and the sanitation system. The fifth key informant was selected in view of her participation in a civil association (Mañaria Bizirik).

### 3.2. Meta-Categories and Thematic Nuclei

Meta-categories and thematic nuclei identified after coding are shown in Table 3.

Each of the thematic nuclei analyzed revealed problems or areas for improvements, health assets, and strengths that may lead to proposals for change. As shown by our results, both the problems and assets identified as well as the proposed actions to address them reflect a broad concept of health, as it is tightly linked to actions that take place in the community, encompassing the range from the public health sector to other sectors like economy, culture, education, or the environment. Problems like the need to work but not being able to find a job, women having to work in addition to carrying out their domestic duties, architectural barriers, lack of resources to support associations, the absence of public transportation, a lack of hygiene in houses, poor access to public spaces, etc., are all health problems because they affect overall well-being and quality of life of the community. When speaking about wellness as part of the health of the community, we must recognize that the public health sector is only one aspect of it, and that other sectors, including structural, social, and political sectors, are often more relevant [36]. The main problems and health assets that were identified by our study included those detailed below.

### 3.3. Meta-Categories

#### 3.3.1. Population

This nucleus included concepts related to problems around housing (high prices, low availability), the need to work outside the community, the transformation of the natural environment caused by the quarry industry, and the lack of services. “It is not that you are not living comfortably, but it is true that there are many things lacking, until eventually you leave” (I-21). Women pointed out that these aspects influenced the demographic development of this community and were strong determinants of community health. These aspects require reflection and searching for a collective way to address them; thus, they go beyond the individual, leading to community and multi-sectorial proposals for change. The proposals for change that were identified by the informants included improvements in the urban sector and the building of new houses. By the end of the project, there were some advances in both, as is shown in Table 4.

#### 3.3.2. From Home to Community Economics

Women identified three kinds of work that contributed both to individual development as well as to personal, domestic, and community economic well-being. (1) Some work is non-remunerated; this is characterized by multiple and varied tasks (visible and invisible) and by personal, family, and social repercussions. It was clear that women were the main actors responsible for strategies related to the reconciliation of work and domestic life. The management positions assumed by women were also clear, underlining the need for socializing these activities in ways that promote other ways of looking at and doing things by all members of the household: “I am the queen of the house, the queen of all jobs” (I-1). (2) Remunerated labor brought with it several psychological and social benefits as well as satisfying basic needs: “I started working this past September, and it has been salvation!” (I-17). (3) Community participation work, known as auzolana, which was accessed by observation and photography, has a strong presence in this community and brings about collaborative and non-remunerated benefits. Among the proposals for action, there was a strong emphasis on strengthening auzolana, leading to the establishment of annual meetings to plan this activity. This proposal was enacted in 2014 and is still in effect. A second proposal requested the participation of citizens in decision making around priorities of local budgets. The proposal was accepted and has been in place ever since.

#### 3.3.3. Public and Private Spaces

Public and private spaces are where daily activities and coexistence take place, and informants considered them clear determinants of health. The rural characteristics of the community were among those values more highly recognized by women, for example, a better quality of life as compared to large cities. However, with respect to public spaces, there were several aspects of the urban landscape that were motive for concern. Some of these are complex, like the quarries, the roads (particularly the main highway that goes across the locality), and problems that certain buildings: “Before, this was like the Alps, a beautiful landscape. Now what? It used to be a nice town, and now you feel like crying, everywhere there is dust, noise …” (I-10). There were also concerns about how to manage residues and the difficulties concerning the need to move to other places to complete some basic daily activities. With respect to private spaces, there were problems and needs related to comfort and accessibility, like peacefulness, safety, crowding, structural maintenance of residential blocks, and coexistence with neighbors. A specific action that was requested was to increase the number of public transportation facets and to expand the schedule between Mañaria and nearby cities. This activity was enacted after December 2018.

#### 3.3.4. Habits and Lifestyles

The topics that emerged in this thematic nucleus provide structure and organization, aspects of daily life for the women who were interviewed. Eating habits are an expression of their beliefs and traditions and are tied to the geographical environment and to food availability. Many inhabitants of this community had a piece of land they cultivated, so there was a positive representation of the daily intake of vegetables and of the custom of having lunch and dinner at home, in the company of other family members. The low consumption of fish compared to meat intake was identified as an area that could improve.

Alcohol consumption was mainly concentrated on weekends and outdoors, as a means of enjoyment, with beer and wine being the preferred drinks.

With respect to smoking, there was a clear group of smokers and ex-smokers that provided an opportunity to analyze and discuss their experiences.

Although most informants said that they slept more than seven hours daily, some expressed problems related to rest, environment, and a disquieting physical or mental state.

With respect to leisure and free time, fun, rest, and personal development, women were aware that these activities contribute to physical, psychological, and social benefits: “Without my bike, I am nervous. … If I am not out in the hill, I ride my bike” (I-6). As such, these practices were carried out on a regular basis for many women.

A specific proposal on this topic had to do with the promotion of physical activity, requesting municipal spaces for public gym practice and for building areas for physical exercise. The first activity was realized in 2014, and the second was approved and enacted in 2019.

#### 3.3.5. Socializing Process

This nucleus identified scenarios that fostered socialization, and hence, personal development among neighbors in this community. Some of these scenarios are formal, like the old school and child-care, while others emerge from feelings of integration, such as those that stemmed from personal relationships and structured socializing in places like church, cultural centers, ball games, the central square, the locales at the town hall, or the retiree house: “When we first got here, it was clear that we had to participate in several things in the community: bars, shops. … Yes, yes!” (I-8). The proposals for change that were requested were meant to improve the integration of the community and enable a healthy socializing process. To achieve this, since 2016 there have been three concrete activities in place: organize leisure-time activities for children within a local social organization; offer space and cultural activities for adolescents; and increase the offer of different activities (informatics, robotics) with a participatory character at the time of budget planning.

#### 3.3.6. Health Care Resources

Many of the women who were interviewed offered informal care to family members living with them, and they were the main resource for other family members in managing health care. There is a positive sense in the way that the elderly are taken care of in the community. The family continues to have a fundamental role in providing care to its members, while at the same time it becomes clear that the social network close to the household, including relatives, neighbors, and friends, are informal care providers. This is illustrated by the case in which a daughter offered informal care and support to her widowed mother for a full decade: “When my mother became a widow, she came to live with us and spent 10 years at home, and all the care she required was provided here. We were supported by health care workers who came almost daily, including the doctor as well as a student; really wonderful!” (I-10). However, this community is no stranger to the feeling of loneliness that afflicts some of its members, as illustrated by this same case, where another informant said: “There are also some elderly persons who live alone, as they are the old guys in town, and they live very much alone… and reject having company, being quite inaccessible, and more weird than weird!” (I-24).

Alongside the informal care system is the formal health care provided by institutions and social entities. Mañaria has a doctor’s office that includes a physician and a nurse. This office provides home visits by the medical staff and treats those requiring services with kindness and efficiency. Some areas need improvement. For example, the following are needed: pediatric services and services for young people in the community; a pediatric continuous care point that is closer to the community than the current one; expanded office hours; a more consolidated and coordinated team; a larger offer of specific procedures, like blood draws; and more community activities.

In addition to the problems and health assets, this study identified several proposals for change, some of which have been enacted at the time of publication, while others are still to be completed (see Table 4). Among the most relevant, since 2014, health education sessions have been offered by health professionals, encompassing different topics. The next objective will be to improve availability and access to a pediatric emergency service in a nearby locality.

**Table 4:** Proposals or solutions identified by the study, including those enacted and those still pending.

Meta Categories	Proposals for Change Identified by Participants (After Feedback and Discussion with the Community)	Proposals for Change, Enacted and Pending (*)
Population	Promote services, improve landscaping and housing	Improvement to the central plaza and its surroundings in Mañaria (based on a project budgeted with participation of children in the community)—January 2017 Inauguration of the Natural Sciences Museum “Hontza Museoa”—October 2014 General Plan for Urban Layout (initially approved in June 2018), considering moderate urban growth involving between 50 and 60 new dwellings (*)
Domestic and community economy	Include younger generations in domestic spaces Foster small businesses Continue supporting auzolana	Opening a hosting business—June 2018 Annual auzolana sessions—2014–2019 Joint participatory budgeting to integrate citizens in official budgeting process in the priority decision-making process—2012–present
Public and private spaces	Promote healthy environments and resources	Increase offer of public transportation to link the community with the cities of Durango and Bilbao—December 2014. Increase frequency of public transportation offer to link the community with the cities of Durango and Vitoria—December 2018
Life habits and lifestyle	Foster physical activity	Begin offering gymnastics exercise for open population—January 2014 Restoration of the ball game (fronton) to increase sports offering to the community—May 2019
Socializing process	Foster community integration Promote individual aspects that lead to a healthy socializing process	A group of parents organized the Andra Mari Association (2016), which organizes activities to allow children to stay in the community, enjoying leisure and free time activities Offer of a cultural space and locale for adolescents (based on a participatory budgeting project that involved youngsters)—2016 Increased offering of capacity-building sessions, including activities such as skating, robotics, bicycle maintenance, social networks, etc.—2017–19
Health care resources	Promote health care processes for men Train agents for informal care Offer pediatric services	Mobilization to restore an emergency pediatrics office in the clinic at Duranguesado—2017 Offer of training sessions to care for back pain, pelvic floor, experiences of death for children, how to address sexuality, first aid for parents and kids—2016–2019.

(*) Proposals marked with an asterix were not yet implemented when the study was concluded.

## 4. Discussion

Our approach to the community diagnosis stems from the concept of the social determinants of health and was deeply rooted in the need to take a comprehensive and multidimensional view of the lives of people living in this community. A second key determinant of our approach, aligned with current trends in primary health care at the community level, was the need to empower women to carry out activities to protect and promote health care, including self-care and fostering dialogue and negotiation between health care institutions and organized women [37,38,39]. While an approach towards a more comprehensive understanding of the health determinants of the community has also been used by other researchers [8,9], what is new in the present study is a merging of the theoretical approach with the relevance and operational potential assigned to female informants, thus making them not only participants but actual leaders in the research. This was achieved by giving women not only a voice as informants, but incorporating them into the discussion of the results, recognizing the value of the nuances uncovered that led to rectification in our understanding of the local situations. This allowed women’s suggestions to be included in a first draft of the study results, when researchers returned to the informants to fine tune and validate their results. At this time, it became evident that what these women had to say and their proposals for actions that could improve the health of the community should be brought to the attention of the whole community as well as to government officials who had decision-making power.

Thus, participants in this study went beyond their role as informants, as they were empowered to be coparticipants in the health diagnosis of the community, which put them in the position to negotiate the will and resources necessary to implement results. We consider this last step as a clear strength of the study, in line with the new gender perspective in primary health care, which tends to empower females for the protection, promotion, and self-care of their own health, fostering dialogue, consultation, and negotiation mechanisms between health care institutions and organized civil society [37,38,39].

Our approach was supported to a large extent by the regional health plan for the Euskadi region (of which Mañaria is part) for the periods 2013–2020 and 2008–2013 [40] and the Department of Health and Osakidetza (the name of the Regional Health Plan in Euskera) in 2013, which highlights the importance of community action in health. This led to the development of the Plan for Comprehensive Health Care for Euskadi [41], which called for a proactive approach to improve the health of the population. Based on these strategies, we conceptualized the community diagnosis, fostering participation of its members, particularly the female population, ensuring active involvement in improving community health, with a broad approach to health determinants. Other key aspects of our strategy included the involvement of local authorities in the overall project, starting early in the process, when researchers informed them of their activities, followed by regular feedback about methods and preliminary results, thus strengthening the credibility of the whole process. Further, there was ample proliferation of the final report, which was presented in oral and written form to the Town Council, including a hard copy to be filed in the community library. This whole process moved the authorities to understand the problems, consider the solutions offered, and implement several of them. Therefore, we believe that the confluence of interests between government officials and female participants, facilitated by the research team, allowed for key proposals for action to be enacted.

Several aspects of the community diagnosis resulted from the specific rural location of this community. Thus, we were able to document and witness the renewed value ascribed to rural settings as potential sites for healthy living, including going back to a natural environment and finding peace and quiet as opposed to the stressful living conditions of urban settings. However, it is important to highlight the drawbacks represented by the absence or limited availability of public services, which represents an obstacle for rural development and demographic recovery. From a political point of view, it is important to promote progressive interventions that may overcome the limitation of basic services for this and similar communities, in response to as well as in support of the proposals that emerge from the population.

Another local aspect that was identified in Mañaria as significantly affecting the health of the community was the quarry industry, which has affected the quality of life of its people for many years. Three different quarries, occupying a significant proportion of the space in the municipality, have been in operation for some time. They generate significant contamination (noise and dirt) as well as disruptions in daily life, while channeling most of the economic benefit outside the community. Activities linked to this industry were perceived as having a strong negative impact on the community, increasing the incentive for the community to sue one of the quarries. The Courts of Justice found several irregularities in the operation of one of the quarry industries (Zalloventa quarry), which led to the cancellation of its activities. Therefore, dismantling one of the active mining activities was perceived as an important step toward general improvement for the community at large.

Two interesting findings in our study highlighted the importance of a sense of community or shared activities by the population. First, it is worth noting that informants placed great importance on public spaces and their relationship to health. Their vision reflects that these spaces may or may not foster the balance and well-being of the individual, strengthening interpersonal relationships and contributing to the promotion or deterioration of the environment. These spaces promote safety (highly valued), tranquility, and trust in daily life. Second, it is worth noting the role played by auzolana or auzolan, which may be defined as a kind of “work performed at the community level, typically on a voluntary basis, with no economic retribution, that seeks a common good by improving communal as well as private spaces, and which may encompass a wide variety of joint tasks” [42]. In our case, auzolana came to be considered an efficient instrument to foster social cohesiveness, leading to the attainment of common goals at a reduced economic cost for the community. While there has been some research published around the efficacy of community activities in health promotion [43,44], we did not find any previous reference related to auzolana and the role it plays as an efficacious way to foster community participation.

From a methodological point of view, coinciding with other authors [13,21], we used qualitative methods for data collection, confirming the usefulness and efficacy of these methods to approach the community health diagnosis. A potential caveat of the ethnographic approach relates to the use of participant observation, which in this case meant that one of the authors (MJA) lived in the community; this may have introduced some bias in the interviews. However, the long time that the researcher spent in the community, as well as her knowledge of the local language, Euskera, may have minimized this risk, as it allowed her to effectively integrate herself into daily living. In addition, participant observation allowed her to have a better context and understanding of several situations, some of them anticipated and some novel, that otherwise would have gone unobserved and unrecorded, limiting their inclusion in the analysis. A second caveat worth mentioning is the long period that it took for the study to be completed. This was due, in large part, to the fact that the study design did not stop at the point of finishing initial data collection to draft the community diagnosis. Instead, it required validation by the participants in the study and the community at large, followed by the identification of proposals for action that could be taken up by the relevant authorities and following through with them. Therefore, by extending the study longer than strictly necessary to elaborate the community diagnosis, we were able to see it through to the implementation phase, effectively linking diagnosis with treatment.

An issue that is open for discussion in our approach is that, by choosing to include only women as informants, we may have missed the point of view of men in the community. Women identified themselves as the main support for providing domestic care, highlighting their dedication to caring for children, the elderly, and those who are sick, while at the same time contributing to household income. Their active community participation points to changes that slowly call for the involvement of men in these activities, especially the younger generation. It is possible that the male point of view might have identified different problems that would have required different solutions. We recognize this bias but were convinced that it was important to understand and adopt the point of view of women, given their relevant roles and experience in promoting and sustaining the health of the community at large. We intentionally sought to incorporate the vision of women in the community to describe and analyze the structural conditionings that bear their influence on the health of the community (employment and work conditions, household and residential environment, values and culture, psychosocial factors, behavioral aspects, health services, informal care network, etc.). These aspects, which have been studied before [45], are the backbone of the current research. Some studies have also included the testimony of women as their main source of data [13,15]. We agree with other authors on the importance of finding models that promote women’s empowerment [46].

This study also helped determine the nature of some of the problems and health assets [45]: healthy environments, available resources, community activities, degree of involvement of specific entities, professionals providing different services with whom it may be possible to work in the future, and so on. While other studies focus on the identification of health assets [45,47], following an approach similar to ours, these have been almost exclusively the center of attention; thus, they do not appear integrated or engrained with other elements of the health diagnosis, as we strived to do in our study. In this sense, the inclusion of health assets in community studies, while novel, clearly shows its utility in contributing to the health diagnosis of the community. Likewise, we have not found previous community health diagnoses that incorporate health assets as part of the data collection.

## 5. Conclusions

In closing, we want to underscore the importance of choosing a qualitative data collection strategy to conduct the community diagnosis, with particular emphasis on a holistic and integrative view of the community, incorporating the specific view of its women as active participants in understanding the problems and proposing solutions. Furthermore, we found great value in going back to the informants to validate our initial findings, in order to refine them and to facilitate looking for potential solutions in the short and medium term, thus turning informants into active participants. These solutions encompassed improvements in health services, environmental improvements, as well as urban and sociocultural changes. We believe that this approach could be reproduced in other settings, contributing to building the experience and knowledge base necessary to scale up these activities.

## Figures and Tables

**Table 1:** Structure of the female population in Mañaria and sample population.

	Structure of the Female Population in Mañaria, by Age Group				Sample Selected for Interview, by Age Group and Zone within the Community (2008)					
						Neighborhood				
Age (years)	2009	2010	2011	Sample	Urban center	Aldebaraieta	Aldebarrena	Aldegoiena	Arrueta	Urkuleta
	*n* (%)	*n* (%)	*n* (%)		15	1	1	1	2	1
10–24	31 (14%)	34 (14%)	31 (13%)	2 (10%)	2	0	0	0	0	0
25–54	102 (46%)	107 (46%)	109 (46)	12 (57%)	8	0	1	1	1	1
55–69	41 (18%)	45 (19%)	48 (20%)	5 (24%)	4	0	0	0	1	0
≥70	37 (17%)	34 (14%)	34 (14%)	2 (10%)	1	1	0	0	0	0
Total	211	220	222	21	15	1	1	1	2	1

**Table 2:** Interview guide for in-depth and structured interviews.

IN-DEPTH (General and Open-Ended Questions)	SEMI-STRUCTURED (Topics)
What do you think about the community’s health in Mañaria?	DEMOGRAPHIC STRUCTURE
What aspects may be involved to make this community more healthy or less healthy?	ECONOMIC STRUCTURE Business and public establishments Industry
What do you like about this community?	URBAN STRUCTURE Solid waste Cleanliness Urban design Urban furniture Housing Green areas Other urban aspects
What things do you miss?	SOCIAL SYSTEM Resources and community services Socializing process
What things do you think should improve?	HEALTH CARE SYSTEM Formal health care system Informal health care system

**Table 3:** Meta-categories and thematic nuclei.

Meta-Categories	Thematic Nuclei	
Population	Housing	
	Work	
	Environment	
Domestic and community economy	Non-remunerated work	
	Remunerated employment	
	Community participatory work	
Public and private spaces	Public space:	Private space:
	Quarries	Comfort
	Main highway	Accessibility
	Architectonic barriers	Tranquility
	Mobility	Overcrowding
	Esthetics	Building maintenance
	Residues	Coexisting with neighbors
	Public safety	Private safety
Life habits and lifestyle	Food	
	Alcohol	
	Tobacco	
	Rest/sleep	
	Leisure and free time	
Socializing process	Formal scenarios:	Informal scenarios:
	Old school	Church
	Childcare	Fronton
		Cultural Center
		Central square
		Retiree House
		Townhall spaces
Health care resources	Care and informal caregivers	Care and formal caregivers: doctor’s office
	Women, main providers	Material resources
	Family	Professional resources
	Neighbors	Services offered
	Social network	House care
		Care

## Data Availability

The data presented in this study are available on request from the corresponding author. The data are not publicly available in deference to participants, who were not informed that their replies, even when de-identified, would be made publicly available.

## References

[1] Ramos E., Darías-Curvo S. (2009). Diagnóstico de salud de la comunidad, métodos y técnicas. Enfermería Comunitaria.

[2] Molewyk M., Ayoola A., Topp R., Landheer G. (2015). Conducting research with community groups. West J. Nurs. Res..

[3] Mason D.J. (2015). Building healthy communities. Nurs. Outlook.

[4] Sousa F.A., Goulart M.J., Braga A.M., Medeiros C.M., Rego D.C., Vieira F.G., Pereira H.J., Tavares H.M., Loura M.M. (2017). Setting health priorities in a community: A case example. Rev. Saude Publica.

[5] Colell E., Sánchez-Ledesma E., Novoa Ana M., Daban F., Fernández A., Juárez O., Pérez K. (2018). El diagnóstico de salud del programa Barcelona Salut als Barris. Metodología para un proceso participativo. Community health assessment of the programme “Barcelona Health in the Neighbourhoods”. Methodology for a participatory process. Gac. Sanit..

[6] Marchioni M., Morin L.M., Álamo J., Buades J., Giménez C. (2013). Metodología de la Intervención Comunitaria. Los Procesos Comunitarios. En: Hagamos de Nuestro Barrio Un Lugar Habitable. Manual De Intervención Comunitaria En Barrios.

[7] Benito A., Nuin B., Sorarrain Y., Blanco M., Astillero M.J., Paskual A., Porta A., Vergara I. (2016). Guía Metodológica Para el Abordaje de la Salud Desde una Perspectiva Comunitaria.

[8] Tamayo M., Besoaín A., Rebolledo J. (2018). Determinantes sociales de la salud y discapacidad: Actualizando el modelo de determinación. Gac. Sanit..

[9] Espelt A., Continente X., Domingo-Salvany A., Domínguez-Berjón M.F., Fernández-Villa T., Monge S., Ruiz-Cantero M.T., Pérez G., Borrel C. (2016). La vigilancia de los determinantes sociales de la salud. Grupo de Determinantes Sociales de la Salud de la Sociedad Española de Epidemiologia. Gac. Sanit..

[10] Escartin P., López V., Ruiz-Jimenez J.L. (2015). La participación comunitaria en salud. Comunidad.

[11] González-González J., Rico-García G., Izaguirre-Zapatera A.M., De Ángel-Larrinaga S., Lor-Martín M. (2016). Mujeres cuidadoras: Intervención comunitaria en mujeres promotoras de salud rural. Med. Gen. Y Fam..

[12] MacCormack C. (1992). Planning and evaluating women’s participation in primary health care. Soc. Sci. Med..

[13] Arenas-Monreal L., Cortez-Lugo M., Parada-Toro I., Pacheco-Magaña L.E., Magaña-Valladares L. (2015). Population health diagnosis with an ecohealth approach. Rev. Saude Pública.

[14] Freidin B., Ballesteros M., Wilner A. (2019). Navigating the public health services: The experiences of women who live in the periphery of Buenos Aires city. Saude Soc..

[15] Li V.C., Shaoxian W., Kunyi W., Wentao Z., Buchthal O., Wong G.C., Burris M.A. (2001). Capacity building to improve women’s health in rural China. Soc. Sci. Med..

[16] Morales I., Navarrete A., Santos C. (1995). La salud de Ronda, percepciones de su gente: Uso de las técnicas cualitativas para conocer el estado. A Tu Salud Rev. De Educ. Para La Salud.

[17] Gobierno de Canarias, Consejería de Empleo y Asuntos Sociales, Ayuntamiento de Telde y Grupo Técnico de Coordinación Las Remudas y La Pardilla (2005). Dos Barrios Hablan. Las Remudas y La Pardilla.

[18] Arenas-Monreal L., Cortez-Lugo M., Parada-Toro I., Pacheco-Magaña L., Magaña-Valladares L. (2015). Diagnóstico de salud poblacional con enfoque de ecosalud. Rev. Saúde Pública.

[19] Ponce M.L., Díaz B., Sánchez B., Garrido M.L., Lara T., Del Ángel de León A., De la Rosa A. (2005). Diagnóstico comunitario de la situación de salud de una población urbano marginada. Vertientes. Rev. Espec. En Cienc. De La Salud.

[20] Ashton J., Grey P., Barnard K. (1986). Healthy cities-WHO’s new public health initiative. Health Promot. Int..

[21] Montaner I., Fernandes S., Badia M., Martinez D., Aranda B., Ruiz F.J., Delgado J., Gonzalez F. (2016). ‘El Carmel’ community-orientation experience. Int. J. Integr. Care.

[22] Sánchez-Ledesma E., Pérez A., Vázquez N., García-Subirats I., Fernández A., Novoa A.M., Davan F. (2018). La priorización comunitaria en el programa Barcelona Salut als Barris. Grupo de Trabajo de Priorización. Gac. Sanit..

[23] Valls-Llobet C. (2008). Salud comunitaria con perspectiva de género. Comunidad.

[24] Spiers J., Morse J.M., Olson K. (2002). Verification Strategies for Establishing Reliability and Validity in Qualitative Research. Int. J. Qual. Methods.

[25] O’Reilly K. (2012). Ethnographic Methods.

[26] Driessnack M., Sousa V., Mendes I.A. (2007). An overview of research designs relevant to nursing: Qualitative designs. Rev. Latino-Am. Enfermagem..

[27] Strauss A.L., Corbin J.M. (2008). Basics of Qualitative Research: Techniques and Procedures for Developing Grounded Theory.

[28] Bernard H.R., Roberts N.E. (2018). Research Methods in Anthropology. Qualitative and Quantitative Approaches.

[29] Gállego-Diéguez J., Aliaga-Traín P., Benedé-Azagra C.B., Bueno-Franco M., Ferrer-Gracia E., Ipiéns-Sarrate J.R., Muñoz-Nadal P., Plumed-Parrilla M., Vilches-Urrutia B. (2016). Las redes de experiencias de salud comunitaria como sistema de información en promoción de la salud: La trayectoria en Aragón. Gac. Sanit..

[30] Balmer C., Griffiths F., Dunn J. (2015). A review of the issues and challenges involved in using participant produced photographs in nursing research. J. Adv. Nurs..

[31] Glaser B.G., Strauss A.L. (2017). The Discovery of Grounded Theory: Strategies for Qualitative Research.

[32] Lancharro-Tavero I., Gil-García E., Macías-Seda J., Romero-Serrano R., Calvo-Cabrera I.M., Arroyo-Rodríguez A. (2018). The gender perspective in the opinions and discourse of women about caregiving. Rev. Esc. Enferm. USP.

[33] World Medical Association (2013). Declaration of Helsinki: Ethical principles for medical research involving human subjects. JAMA.

[34] Tong A., Sainsbury P., Craig J. (2007). Consolidated criteria for reporting qualitative research (COREQ): A 32-item. Checklist for interviews and focus groups. Int. J. Qual. Health Care.

[35] Calderón C. (2002). Quality criteria in qualitative health research: Notes for a necessary debate. Rev. Esp. Salud. Pública.

[36] Sobrino K., Hernán M., Cofiño R. (2018). ¿De qué hablamos cuando hablamos de «salud comunitaria»?. Gac. Sanit..

[37] Martin-García M., Ponte-Mittelbrun C., Sánchez-Bayle M. (2006). Participación social y orientación comunitaria en los servicios de salud. Gac. Sanit..

[38] World Health Organization (1988). Jakarta Declaration on Health Promotion in the 21st Century. Proceedings of the Fourth International Conference on Health Promotion.

[39] World Health Organization (2000). Declaration of Mexico towards greater equity. Proceedings of the Fifth International Conference on Health Promotion towards greater equity.

[40] Gobierno Vasco (2013). Osasuna, Pertsonen Eskubidea, Guztion Ardura. Políticas de Salud para Euskadi 2013–2020.

[41] Departamento de Salud, Sede Web, Gobierno Vasco (2016). Plan de Atención Integrada en Euskadi. http://goo.gl/1bfLGI.

[42] Saenz del Castillo A. (2019). De las Hermandades a la Seguridad Social. Estudios sobre previsión social en el País Vasco, siglos XIX–XXI. Hist. Contemp..

[43] March S., Soler M., Miller F., Montaner I., Pérez M.J., Ramos M. (2014). Variabilidad en la implantación de las actividades comunitarias de promoción de la salud en España. An. Sist. Sanit. Navar..

[44] Leon M., Jimenez M., Vidal N., Bermudez K., De Vos P. (2020). The Role of Social Movements in Strengthening Health Systems: The Experience of the National Health Forum in El Salvador (2009–2018). Int. J. Health Serv..

[45] Cofiño R., Aviñó D., Benedé C., Botello B., Cubillo J., Morgan A., Paredes-Carbonell J.J., Hernán M. (2016). Promoción de la salud basada en activos: ¿cómo trabajar con esta perspectiva en intervenciones locales?. Gac. Sanit..

[46] Kar S.B., Pascual C.A., Chickering K.L. (1999). Empowerment of women for health promotion: A meta-analysis. Soc. Sci. Med..

[47] Sánchez-Casado L., Paredes-Carbonell J.J., López-Sánchez P., Morgan A. (2017). Mapa de activos para la salud y la convivencia: Propuestas de acción desde la intersectorialidad. Index. Enferm..

